# Are Dietary Patterns of Mothers during Pregnancy Related to Children's Weight Status? Evidence from the Lifeways Cross- Generational Cohort Study

**DOI:** 10.3934/publichealth.2015.3.274

**Published:** 2015-07-23

**Authors:** Celine M Murrin, Mirjam M Heinen, Cecily C Kelleher

**Affiliations:** 1 School of Public Health, Physiotherapy, and Sports Science, University College Dublin, Woodview House, Belfield, Dublin 4, Ireland

**Keywords:** dietary patterns, prospective cohort, pregnancy, mothers, fathers, child obesity

## Abstract

Children's diet patterns are likely to be influenced by their mothers' diet pattern. The primary objective of this study was to examine whether children's adiposity could be influenced by diet patterns of mothers during pregnancy. A secondary objective was to study the relative influence of fathers' and children's dietary patterns on childhood adiposity. The design was a prospective cross-generational cohort study initiated with 1,124 mothers recruited during pregnancy. Self-reported questionnaires included a food frequency instrument (FFQ) to assess parental intakes during the perinatal period. Child body mass index (BMI) was measured at 5 years and an age-appropriate FFQ was administered. Dietary patterns for each group were identified by principal components analysis. Pearson's correlation and logistic regression were used to test for associations. Dietary patterns were described for *n* = 1,042 mothers during pregnancy and *n* = 331 fathers during the perinatal period. Dietary patterns and BMI data were available for *n* = 443 children at age 5 years. The diet patterns identified for mothers correlated with the corresponding diet patterns for fathers. The children's “pasta & vegetable” pattern was positively correlated with “healthy patterns” in mothers (*r* = 0.195, *p* < 0.01) and fathers (*r* = 0.250, *p* < 0.01). The children's “junk” food pattern was correlated with the “processed” pattern in mothers (*r* = 0.245, *p* < 0.01) and fathers (*r* = 0.257, *p* < 0.01). In multivariate logistic regression analysis the upper tertiles of children's “cereal and juice” [Tertile 2 (T2): OR 0.44, 95% CI (0.22–0.90); T3: 0.41, (0.19–0.85)] and the middle tertile of the “pasta and veg” patterns [T3: 0.37, (0.18–0.75)] were negatively associated with overweight and obesity. The mothers' processed pattern during pregnancy was positively associated with offspring overweight and obesity [T2: 2.64, (1.28–5.45); T3: 2.03, (0.87–4.73)]. No significant associations were observed for the paternal diet patterns. This analysis shows that the influence of maternal diet pattern on child obesity is apparent early in the lifecourse.

## 1. Introduction

The concept of early life environmental factors relating to later disease originated towards the end of the last century, with a particular focus on the role of nutrition status during pregnancy and the advent of the foetal origins hypothesis [1],[2]. The exploration of this phenomenon has largely evolved from quasi-experimental studies [3],[4] and dietary manipulation of pregnant animal models [5]–[8]. Furthermore, a growing body of evidence has highlighted how some of these early life exposures, not only influence offspring of the first generation but may be transmitted to future generations [9]–[11].

Prospective human cohorts allow for habitual diet during pregnancy to be explored and many cohorts are established with the purpose of identifying dietary exposures and health-related outcomes. Diet during pregnancy has been explored by focusing on specific food items including seafood and fish intakes [12]–[14], fruit and vegetables [15],[16], and oils [17]. More recently, the patterns of food consumption have been considered, as they not only provide greater understanding of the way diet affects disease, but also provide for clearer public health messages to improve diet overall. One of the first studies to assess dietary patterns during pregnancy was in a population of Mexican-American women [18]. Multivariate analysis of the dietary patterns with offspring birth weight indicated that nutrient dense diet patterns that were either high in meats or dairy products or high in fruits and vegetables and low in dairy products were associated with increased birth weight. In contrast, patterns high in fats and oils, breads and cereals, high fat meats, and sugar were associated with decreased birth weight [18]. Analysis of specific dietary patterns has been conducted using an *a priori* approach, including patterns based on the Healthy Eating Index, the DASH diet, and the Mediterranean diet [19]–[21].

Research on dietary patterns during pregnancy has been explored with a range of health outcomes for both the mother and her offspring. Maternal outcomes include diet quality [22], nutrient intake [23]–[25], gestational weight gain [26] cardiovascular risk factors [27], and depression [28]. The child outcomes include congenital defects [29],[30], wheeze [31]–[33], bone mass and fractures [34],[35], and child behavior [36]. Other large birth cohorts have reported associations between diet patterns and preeclampsia and preterm births [37]–[39]. One of the largest birth cohorts, the Danish National Birth Cohort, described three major diet patterns in their cohort of pregnant women and found the mothers consuming a “Western” diet were more at risk of delivering a small for gestational age infant than those consuming other diets [40]. Foetal growth was also explored with one study demonstrating positive associations for energy-rich dietary patterns [41].

In Britain, dietary patterns during pregnancy have been described by two cohort studies [24],[42] but neither have yet reported associations with childhood body composition. Diet patterns in children have been described in other cohort studies [43]–[46] and a recent review concluded that childhood dietary patterns consisting of energy dense, high fat and low fibre foods were positively associated with obesity [47].

Lifestyle factors, which increase the risk of obesity, tend to cluster in families [48]. The Avon Longitudinal Study of Parents and Children has reported that certain diet patterns tend to be similar in couples [49]. Studies have reported on the associations with children's patterns and the influence of their parents [50]–[52]. Fisk et al. have reported on maternal influences on dietary patterns in three year old children and found that children scoring highly on a prudent diet pattern were more likely to have mothers with similar prudent diet patterns [53]. Studies of diet and child obesity have tended to focus on maternal influences with limited evidence available for the father's diet. However, a recent animal model has highlighted the potential for paternal programming of obesity [54].

To our knowledge, the influence of diet pattern during pregnancy on offspring body composition has not been studied or reported elsewhere. The primary objective of this study was to describe the diet patterns of mothers during pregnancy, using a longitudinal birth cohort, and examine whether certain patterns were independently associated with offspring adiposity at the age of five. A secondary objective was to examine whether there were any associations with the father's diet pattern during the perinatal period. Cross-sectional diet patterns in the children at age five were considered in both explorations.

## 2. Methods

The Lifeways study is a prospective cohort study which was established between 2001 and 2003. A total of 1,124 pregnant women from 2 maternity hospitals in Ireland were recruited at the time of their first ante-natal visit [55]. At this visit, a midwife invited Irish-born mothers to participate and, following the provision of informed consent, the women completed a questionnaire relating to general health, lifestyle, diet and socio-demographic characteristics. Partners were invited to participate in the study by the women who consented and provided their partner's contact details. The partners completed a similar questionnaire following the ante-natal visit.

There were 1,082 mothers with live births in Lifeways and all of these were invited to participate in the follow-up when their children were aged five years on average. A sample of 669 (62%) mothers agreed to participate; *n* = 585 agreed to their child being measured for height and weight. Anthropometric measurements were conducted in the home by a trained researcher. Heights and weights were measured to the nearest 0.1 cm and 0.1 kg using standardized protocols [56],[57]. Body mass index was calculated and children were classified as overweight/obese if their BMI exceed the 85^th^ centile (z score 1.036) of the UK BMI reference data 1990 [58],[59].

### 2.1. Dietary assessment

A Food Frequency Questionnaire (FFQ) was administered to mothers to report the frequency of intake of 149 food items since they became pregnant or during the first trimester of pregnancy [60]. Fathers completed the same FFQ, reporting on their average consumption over the previous year. The FFQ was based on the European Prospective Investigation into Cancer and Nutrition instrument [61] where the average frequencies were “never or less than once per month”, “1–3 per month”, “once a week”, “2–4 per week”, “5–6 per week”, “once a day”, 2–3 per day”, “4–5 per day” and “6+ per day”. The amounts were considered as medium servings or common household units for each food and later converted to quantities (grams per day) using standard portion sizes.

Parents completed a FFQ for their child when they were five years of age. This FFQ was developed specifically for the Lifeways study and, since at that time there was no detailed information on dietary intakes of five year old Irish children, a food list was derived from the UK National Diet and Nutrition Survey (NDNS) of 1½–4½ year old children [62]. This instrument was chosen for children because the ages examined were similar to those in Lifeways and, compared to other international studies, the foods listed were more representative of commonly consumed Irish foods. The list has fewer items [52] than the adult list; a shorter list was considered appropriate for this demographic as young children do not have the same variety in their diets as adults and they tend to eat the same kind of foods repeatedly [63],[64]. Secondly, the appearance of different food lists may minimize the repetition in response as mothers may be inclined to complete the FFQ with the same responses as for the mothers' self-completed questionnaire at follow-up. It includes foods particular to children's diets which can provide a pattern in terms of obesity risk [65] and also foods which have nutrients of interest [66]. At the time of development, no standard portion sizes were available for children, therefore portion size data from the UK Food Standards Agency were used which were derived from the UK NDNS [67]. These portion sizes were used to convert the food items to quantities (grams) per day.

### 2.2. Dietary patterns

Dietary patterns of mothers and fathers during pregnancy (baseline) and children at the age of five years were defined using principal components analysis (PCA), an *a posteriori* data reduction technique which identifies underlying patterns in food intake. The original list of 149 food items from the adult FFQ was reduced to 40 food items by grouping food items together which were similar in composition or nutrient value (for instance Crackers were included with “white bread”). The food groups for the analysis were based on those published by the Southampton Women's Study (SWS) which also examines diet in pregnancy, in a sample similar to the Lifeways population, using a FFQ instrument [42].

The original children's FFQ was comprised of 52 food items which were regrouped to resemble as closely as possible that of the mother. However, certain food items were not included in the child's FFQ (such as wine, spirits, tea and coffee); therefore the final number of food groups used for diet pattern analysis was 30.

Principal components analysis was used to derive food patterns based on the 40 parent food groups. The factors were orthogonally transformed by using varimax rotation to maximize the dispersion of loadings within factors while ensuring they are independent. The Kaiser-Meyer-Olkin measure was used to verify the adequacy of the sample (KMO = 0.732). An initial analysis was conducted to obtain eigen values for each component in the data. A total of fourteen dietary patterns were obtained with eigenvalues > 1.0 and, in combination, explained 55.5% of the variance. The analysis was repeated while extracting factors with eigenvalues > 1.4 based on a scree plot. This resulted in 6 factors which explained 32.7% of the variance that were used for subsequent analysis. For the fathers' PCA the eigen value was set at 1.5 which resulted in 6 factors which explained 35% of the variance. Foods with factor loadings > 0.3 were used to define the diet pattern, although foods with loadings ranging from 0.2 to 0.3 were also considered. Diet pattern scores were derived from the sum of standardized daily intake of each food group multiplied by the factor loading for each group using the Anderson-Rubin method in SPSS which produces factor scores that are uncorrelated and standardised. A high factor score for a given pattern indicated high intake of the foods constituting that food pattern and a low score indicated low intake of those foods [68]. The food patterns were labeled by the main food types found within that group and are merely illustrative as opposed to an exact description of the pattern. The proportion of variance explained by the dietary pattern score was calculated for individual foods by summing the squared correlations for each food.

The principal components analysis procedure, as described for the parents, was also employed for the diet pattern analysis of the child. Children with individual food items missing were excluded from the analysis (*n* = 15) resulting in a final sample of *n* = 52. The Kaiser-Meyer-Olkin measure was used to verify the adequacy of the sample (KMO = 0.659). An initial analysis was conducted to obtain eigen values for each component in the data. A total of twelve dietary patterns were obtained with eigenvalues > 1.0 and in combination explained 60.2% of the variance. The analysis was repeated while extracting factors with eigenvalues > 1.5 based on a scree plot. This resulted in 5 factors which explained 33.4% of the variance that were used for subsequent analysis. Foods with factor loadings > 0.3 were used to define the diet pattern although foods with loadings ranging from 0.2 to 0.3 were also considered. Diet pattern scores are derived in the same manner as for the parental diet.

### 2.3. Statistical analysis

The relationship between the diet pattern component scores of the family triad were tested using Pearson's correlation co-efficient. Scores for each family member were subsequently divided into tertiles and used to estimate the odds of overweight/obese in the children at age five years. Initially, univariate logistic regression analysis was employed for each dietary component; the components were subsequently entered into the same model. A series of models was then generated to identify whether diet patterns in either parent during the perinatal period (considered as the baseline) or child at age five years were associated with overweight/obesity, while adjusting for energy intake (kcal), birthweight, parental BMI, age, smoking, and educational status. Odds ratios and 95% CI were used to test the strength of the associations.

### 2.4. Ethics

Ethical approval for the Lifeways Cross-Generation Study was granted by the National University of Ireland, Galway; The Coombe Women's Hospital, Dublin; University College Hospital, Galway; The Irish College of General Practitioners. Approval for the 2007–2008 follow-up study was granted by the Human Research Ethics Committee in University College Dublin

## 3. Results

Dietary information at five years was available for *n* = 567 children and BMI data was available for *n* = 551. Following data cleaning and removal of implausible values, a sample of *n* = 443 children had dietary data with matched BMI at age five years. Of the *n* = 1082 mothers with questionnaire data, maternal dietary information during pregnancy was available for *n* = 1042 mothers. When matched with children's weight and diet measures at age five the sample available for analysis was *n* = 435 mother-child pairs. A sample of *n* = 348 fathers answered the baseline questionnaire (during their partner's pregnancy), n = 331 provided dietary information and n = 191 of their children had dietary information and were measured at age five years.

Socio-demographic differences were noted between the mothers whose children participated in the follow-up (Table 1). The mothers who participated were more likely to be non-smokers, more educated and older in age. However, there were no significant differences observed between the groups for the mother's BMI pre-pregnancy or energy intakes during pregnancy. No significant differences were observed for the fathers whose children participated in the follow-up.

**Table 1.** Differences in characteristics at baseline between parents whose children were measured and children that were not measured at five years.

	Mother with Child's BMI Data at 5 years					Father with Child's BMI Data at 5 years		
	Total		Yes	No		Total	Yes	No
	(*n* = 1082)		(*n* = 551)	(*n* = 531)		(*n* = 348)	(*n* = 232)	(*n* = 116)
Baseline Variables			*n*	*n*			*n*	*n*
Smoker			(%)	(%)			(%)	(%)
No	75.40%		436 (79.3%)	380 (71.6%)		71.80%	169 (73.5%)	80 (68.4%)
Yes	24.50%		115 (20.8%)	151 (28.4%)		28.20%	61 (26.5%)	37 (31.6%)
	χ^2^ = 8.64	*				χ^2^ = 0.996		
Education								
Third level	49.60%		292 (52.7%)	241 (46.3%)		51.40%	124 (53.7%)	54 (47.0%)
Up to second level	50.40%		262 (47.3%)	279 (53.7%)		48.60%	107 (46.3%)	61 (53.0%)
	χ^2^ = 4.34	*				χ^2^ = 1.39		
Age								
>33	32.70%		210 (38.1%)	146 (27.1%)		33.30%	79 (34.1%)	37 (31.9%)
28 to 33	33.10%		190 (34.4%)	171 (31.8%)		33.30%	84 (36.2%)	32 (27.6%)
<28	34.20%		151 (27.4%)	221 (41.1%)		33.30%	69 (29.7%)	47 (40.5%)
	χ^2^ = 24.87	**				χ^2^ = 4.53		
BMI Group								
Normal	71.90%		354 (72.0%)	326 (71.8%)		34.50%	65 (34.8%)	31 (34.1%)
Overweight	19.80%		100 (20.3%)	87 (19.2%)		50.40%	96 (51.3%)	44 (48.4%)
Obese	8.40%		38 (7.7%)	41 (9.0%)		14.10%	26 (13.9%)	16 (17.6%)
	χ^2^ = 0.645					χ^2^ = 0.665		
Mean Energy Intake (SD) kcal	2454 (775)		2419 (750.2)	2490 (799.4)		2560(904)	2515 (827.7)	2647 (1033)
	t = 1.48					t = 1.26		

χ^2^ test for significant difference between groups; t-test for difference in energy intake; †*p* < 0.1, *p < 0.05, **p < 0.01.

Six dietary components were identified using principal components analysis and used to best describe the food intake patterns of the Lifeways mothers during their pregnancy and fathers at baseline. The six components explained 32.7% and 36.3% of the variance respectively. In Table 2 the factor loadings for the components are presented for mothers and fathers simultaneously as the patterns were extracted in a similar order.

**Table 2.** Factor loadings of the food groups in six principal dietary components (patterns) identified for mothers during their pregnancy and identified in fathers in the year preceding their partner's pregnancy (baseline).

Diet pattern	*Healthy*			*Processed*			*Cereal & Fruit*			*Tea & Bread*			*Traditional*			*Confectionery*	
	Mothers	Fathers		Mothers	Fathers		Mothers	Fathers		Mothers	Fathers		Mothers	Fathers		Mothers	Fathers
(variance explained)	(7.0%)	(7.2%)		(5.8%)	(6.8%)		(5.7%)	(6.1%)		(5.2%)	(5.5%)		(5.1%)	(4.9%)		(3.8%)	(4.8%)
Salad vegetables	**0.620**	**0.318**		−0.049	0.176		0.175	**0.356**		0.083	−0.160		−0.037	***0.232***		−0.195	0.031
Other vegetables	**0.610**	**0.679**		−0.181	−0.038		0.083	0.181		0.011	−0.027		−0.103	0.054		−0.086	−0.024
Rice & Pasta	**0.575**	**0.598**		−0.001	0.116		***0.243***	***0.298***		−0.160	0.037		0.003	−**0.227**		0.005	−0.104
Salad oils	**0.501**	**0.332**		0.013	−0.005		0.085	0.143		***0.214***	0.118		−0.034	0.072		−-0.010	0.177
Oily & shell fish	**0.477**	**0.350**		−***0.239***	0.026		0.092	0.189		0.017	*0.232*		−0.012	0.150		0.161	−0.055
Cheese	**0.446**	**0.353**		−0.063	0.157		0.182	0.158		***0.249***	**0.351**		−***0.231***	0.028		0.087	−0.113
Quiche	**0.367**	**0.389**		0.091	0.085		−0.039	0.002		−0.121	0.036		0.138	−0.057		−0.119	−0.016
Chicken	**0.333**	0.044		0.119	**0.429**		−0.007	0.130		−0.047	−0.039		−0.017	−***0.212***		0.096	−0.033
Soft drinks	−0.069	−0.016		**0.650**	**0.622**		0.108	−***0.205***		0.029	−0.072		−0.076	−0.027		−0.005	0.124
Chips & roast potatoes	−0.006	−0.179		**0.613**	**0.534**		−0.090	−0.021		0.105	0.075		0.195	**0.326**		−0.049	0.073
Crisps	0.031	0.078		**0.576**	**0.690**		0.032	−0.056		0.090	0.093		0.109	0.152		0.093	0.076
Fibre cereals	0.137	0.194		−***0.410***	−*0.217*		**0.406**	**0.450**		0.067	−0.036		0.137	−0.057		0.148	−0.065
Pizza	**0.317**	0.128		**0.359**	**0.569**		−0.078	0.016		0.036	0.059		0.027	0.020		0.162	−0.146
Citrus and other fruits	0.103	0.093		−0.038	−0.011		**0.650**	**0.675**		−0.079	−0.125		0.060	0.010		−0.119	0.151
Fruit	0.098	0.070		−0.078	−0.131		**0.592**	**0.694**		0.014	−0.029		0.091	−0.013		−***0.211***	0.163
Yoghurts	0.193	0.112		0.076	−0.019		**0.474**	**0.440**		0.033	0.047		−-0.173	−0.011		0.113	0.008
Fruit juice	0.126	**0.315**		0.119	0.139		**0.423**	0.062		0.015	−0.102		−0.066	−0.008		0.092	0.155
Beer & spirits	***0.273***	***0.265***		−0.039	0.152		−**0.352**	−***0.245***		0.126	0.081		0.115	***0.252***		0.117	−***0.254***
Dried & tinned fruit	0.033	−0.012		−0.108	0.118		**0.336**	**0.497**		0.080	0.081		0.143	0.050		0.183	−0.162
Puddings	0.009	0.020		***0.207***	0.181		**0.326**	0.002		0.081	−0.054		***0.259***	0.105		**0.300**	**0.692**
White bread	−0.073	−*0.218*		***0.261***	0.112		−0.008	0.121		**0.613**	**0.669**		−0.138	0.119		−0.014	−0.021
Full fat spread	0.002	0.006		0.049	0.062		0.005	−0.077		**0.587**	**0.553**		0.076	**0.322**		0.067	0.023
Tea & coffee	0.081	0.076		−0.128	−*0.236*		−***0.222***	−0.007		**0.492**	**0.667**		0.139	−0.074		0.093	0.080
Sugar	0.030	0.034		0.136	0.057		0.002	−0.085		**0.482**	**0.561**		0.040	−0.120		0.059	0.114
Wholemeal bread	0.135	**0.367**		−***0.311***	−**0.304**		***0.297***	0.030		**0.390**	−0.040		−0.074	***0.277***		−0.050	**0.305**
Eggs and egg products	***0.276***	0.048		0.141	0.128		0.037	−0.106		**0.302**	−0.020		***0.269***	***0.244***		−0.089	0.042
Other cereals	−0.168	−0.118		0.190	0.096		0.105	0.112		0.193	0.034		0.066	−0.079		−0.004	**0.481**
Red meat	0.047	−0.079		0.150	−0.006		0.015	−0.070		−0.001	0.010		**0.580**	**0.546**		0.162	−0.068
Full fat milk	−0.182	−*0.256*		−0.044	0.043		0.053	−0.057		0.124	0.083		**0.557**	0.172		0.004	0.077
Processed meat	0.021	−0.118		**0.394**	**0.384**		0.051	−0.098		0.116	−0.029		**0.442**	**0.403**		0.038	***0.274***
Other milk	0.068	0.128		−0.090	−0.019		0.080	***0.229***		0.049	0.050		−**0.419**	−0.196		−0.007	***0.204***
Boiled potatoes	−0.087	−0.141		−0.114	−0.073		0.074	0.130		**0.357**	*0.202*		**0.402**	**0.668**		−0.144	0.015
White fish	***0.290***	0.027		−0.025	0.087		0.045	***0.211***		0.002	0.013		**0.388**	**0.300**		0.087	−0.001
Beans & pulses	***0.262***	**0.630**		0.119	−0.155		0.155	0.181		0.165	−0.104		***0.286***	0.184		−***0.284***	0.064
Reduced fat spread	0.034	0.059		0.028	−0.075		***0.215***	***0.283***		***0.246***	0.055		−***0.259***	0.071		0.006	***0.238***
Cakes & biscuits	−0.058	0.139		0.108	0.011		0.203	−0.030		0.199	0.137		0.122	0.074		**0.590**	**0.696**
Sweets & chocolate	−0.083	−0.049		**0.411**	**0.634**		0.089	−0.052		0.140	0.006		0.072	−-0.002		**0.481**	**0.389**
Wine	***0.249***	**0.468**		−0.080	−0.085		−0.119	−0.138		−0.017	0.015		0.078	−0.107		**0.462**	0.009
Vegetables	**0.320**	***0.294***		−0.165	−0.145		***0.233***	***0.298***		0.141	−0.065		***0.259***	**0.578**		−**0.338**	0.071
Soups & Sauces	0.178	***0.275***		0.137	−0.065		***0.225***	0.012		***0.247***	−***0.228***		0.143	***0.269***		−***0.293***	0.115

Loadings above 0.3 are shown in bold; loadings between 0.2 and 0.3 are shown in bold italics.

The first component was comprised of high loadings from salad vegetables, other vegetables, rice, pasta, salad oils, oily and shell fish, cheese, quiche, vegetables and was therefore labeled “healthy”. The second component was described as “processed” as it indicated consumption patterns of soft drinks, chips, roast potatoes, crisps, pizza, processed meat, sweets and chocolate with negative associations for wholemeal bread and fibre cereals. The third component yielded high loadings on fibre cereals, citrus, and other fruits, yoghurts, fruit juice, dried and tinned fruit, puddings, and wholemeal bread and was therefore described as “cereal & fruit”. The fourth component was labeled “tea & bread” as it contains high loadings of white bread, full fat spread, tea and coffee, and sugar (jam). The fifth component was typical of “traditional” Irish diets with high intakes of red meat, full fat milk, processed meat (hams, sausages, etc.), boiled potatoes, white fish (coated fish) and vegetables. The final component was comprised of puddings, cakes and biscuits, sweets and chocolate, therefore described as “confectionery”.

The principal components analysis of the children's diet revealed five dietary patterns (Table 3). The first pattern was comprised of a range of different foods – fruit, vegetables, yoghurt, wholemeal bread, cheese, eggs, and potatoes and was therefore described as “healthy”. The second component was described as “junk” with high factor loadings for puddings, chips and roast potatoes, sweets and chocolate, and soft drinks. High loadings for red meat, processed meat, chicken, cakes and biscuits, white bread and pizza were identified as “processed”. The next component had the highest loadings for vegetables, salads, rice and pasta and was therefore labelled “pasta and veg”. Finally, the last group had high loadings for fruit juice and other cereals and was identified as “cereals and juice”.

The correlations between the diet pattern scores obtained from family triads are shown in Table 4. Each diet pattern for the mothers correlated with corresponding diet pattern for the fathers. Significant positive correlations (*r* > 0.200) are reported. The “junk” diet pattern for children was consistently correlated with the processed pattern in both parents (M: *r* = 0.245, *P* < 0.01; F: *r* = 0.257, *P* < 0.01), while the pasta & veg pattern was correlated with the healthy pattern in both parents (M: *r* = 0.195, *P* < 0.01; F: *r* = 0.250, *P* < 0.01). The mothers' healthy pattern was correlated (*P* < 0.01) with the fathers' (*r* = 0.316) and children's (*r* = 0.202) healthy pattern. Mothers' traditional pattern was correlated with the children's processed patterns (*r* = 0.233) and the fathers' traditional pattern (*r* = 0.208).

Associations between tertiles of diet patterns of family members and child overweight/obesity are shown in Table 5. The proportions of overweight and obesity across the tertiles of diet scores did not show clear trends. Children in the highest tertile for the healthy diet pattern had a higher proportion of overweight/obesity relative to those in the lowest tertile (37.1% versus 29.8%). Conversely, children in the highest tertile for the junk pattern had the lowest proportion of overweight/obesity (30.4% versus 36.1%). When all the diet patterns were adjusted together in a multivariate analysis, only the children's cereal and juice pattern was significantly negatively associated with overweight/obesity in children. In the first fully adjusted model, which also adjusted for the mothers' diet patterns in pregnancy, the association for the cereal and juice pattern remained [Tertile 2 (T2): Odds Ratio (OR): 0.44, 95% Confidence Interval (CI) 0.22–0.90]; T3: OR 0.41, 95% CI 0.19–0.85). In addition, there was a negative association with the children's pasta and vegetable pattern (T2: OR 0.36 95% CI 0.18–0.75; T3: OR 0.77, 95% CI 0.37–1.62). However, associations with the healthy, junk and processed patterns were not significant. This fully adjusted model also showed a strong and significant association for the mothers' processed pattern (T2) during pregnancy; the highest tertile was also positive but not significant (T2: OR 2.64, 95% CI 1.28–5.45; T3: OR 2.03, 95% CI 0.87–4.73). The mothers' traditional pattern was negatively associated with overweight and obesity. When adjusted with the fathers' diet patterns, the children's cereal and juice pattern remained significantly associated with overweight and obesity (T2: OR 0.12, 95% CI 0.02–0.72; T3: OR 0.07, 95% CI 0.01–0.42). The fathers' diet patterns were not significantly associated with the children's weight status at the age of five.

**Table 3.** Factor loadings of the food groups in five principal dietary components (patterns) identified for children at the age of five.

Child's Diet Pattern	*Healthy*	*Junk*	*Processed*	*Pasta & veg*	*Cereals & juice*
(Variance explained)	(7.24%)	(6.72%)	(5.9%)	(5.8%)	(5.14%)
Fruit	**0.678**	−0.015	−0.057	0.048	−0.015
Citrus & other fruit	**0.665**	−0.065	−0.020	0.170	0.067
Vegetables	**0.647**	0.103	0.136	0.005	−***0.245***
Yoghurt	**0.409**	***0.253***	0.030	−0.097	−0.173
Wholemeal bread	**0.400**	−0.169	0.178	0.031	**0.311**
Cheese	**0.378**	0.034	−0.019	0.174	0.087
Eggs & egg product	**0.303**	0.069	0.123	***0.289***	−0.006
Puddings	−0.017	**0.702**	0.000	0.071	−0.131
Chips & roast potatoes	0.000	**0.686**	0.015	0.197	0.052
Sweets & chocolate	−0.013	**0.621**	0.144	−0.177	0.061
Soft drinks	0.152	**0.499**	***0.216***	−0.137	0.126
Red meat	0.108	−0.075	**0.575**	0.124	−***0.230***
Processed meat	−0.161	***0.274***	**0.522**	0.052	−0.145
Chicken	0.033	0.160	**0.499**	0.089	−0.014
Cakes & Biscuits	0.128	***0.274***	**0.446**	−0.057	***0.281***
White bread	−0.128	***0.226***	**0.420**	−0.081	0.152
Other milk	0.068	−0.140	**0.412**	−0.104	0.044
Pizza	−0.081	0.020	**0.378**	***0.240***	−0.043
Full fat spreads	0.105	0.122	**0.326**	−0.051	0.054
White fish	0.163	−0.060	**0.312**	0.149	−0.147
Salad vegetables	***0.272***	−0.057	0.008	**0.638**	−0.029
Other vegetables	0.161	0.098	−0.118	**0.637**	−0.183
Rice & Pasta	0.027	−0.047	0.160	**0.526**	0.007
Boiled potatoes	**0.395**	−0.003	0.203	−**0.445**	−***0.211***
Salad oils	0.031	−0.078	***0.296***	**0.323**	0.133
Other cereals	−0.084	0.173	0.086	0.097	**0.559**
Fruit juice	0.198	0.197	−0.114	0.022	**0.495**
Fibre cereals	0.110	0.124	0.073	0.113	−**0.453**
Beans and pulses	0.109	***0.247***	0.063	***0.257***	−**0.373**
Low fat spreads	***0.228***	−0.032	0.127	0.143	***0.232***

Loadings above 0.3 are shown in bold; loadings between 0.2 and 0.3 are shown in bold italics.

**Table 4.** Correlation coefficients between child diet patterns aged five and maternal and paternal diet patterns during pregnancy (baseline).

	*Child Pattern*										*Maternal Pattern*										
	Healthy_C_		Junk_C_		Processed_C_		Pasta & Veg._C_		Cereal & Juice_C_		Healthy_M_		Processed_M_		Cereal & Juice_M_		Tea & Bread_M_		Trad'l_M_		Conf'y_M_
*Maternal Pattern*	(*n* = 435)																				
Healthy_M_	0.202	**	−0.043		0.134	**	0.195	**	0.121	*											
Processed_M_	−0.032		0.245	**	0.084		−0.128	**	0.074												
Cereal & Fruit_M_	0.147	**	−0.039		−0.066		0.064		0.018												
Tea & Bread_M_	−0.002		0.076		0.047		−0.049		0.063												
Traditional_M_	0.041		0.077		0.233	**	−0.105	*	−0.052												
Confectionery_M_	−0.077		−0.033		0.144	**	−0.051		0.148	**											
*Paternal Pattern*	(*n* = 192)										(*n* = 312)										
Healthy_F_	0.126		−0.186	**	0.093		0.250	**	0.083		0.316	**	−0.146		0.061		−0.117	*	−0.019		0.008
Processed_F_	−0.053		0.257	**	0.065		−0.009		0.059		0.086		0.193	**	−0.043		0.039		−0.013		−0.006
Cereal & Fruit_F_	0.021		−0.082		0.080		0.187	**	−0.039		−0.038		−0.200	**	0.213	**	0.042		−0.050		−0.071
Tea & Bread_F_	−0.076		−0.016		0.085		−0.157	*	−0.068		−0.030		−0.096		−0.002		0.168	**	−0.008		0.043
Traditional_F_	−0.044		0.141		0.074		−0.025		−0.162	*	−0.114	*	0.144	*	0.018		0.168	**	0.208	**	−0.096
Confectionery_F_	0.025		0.011		0.011		0.088		0.039		0.028		−-0.066		0.017		0.039		−0.037		0.013

C, M, F denote Child, Mother, Father respectively; Trad'l: Traditional; Conf'y: Confectionery; Significant correlations are denoted by:*p < 0.05, ** *p* < 0.01

**Table 5.** Tertiles of dietary pattern scores for children aged five on predicting overweight among Irish children aged 5 years and adjusted for mothers' and fathers' diet patterns at baseline.

						Normal v Overweight/obese (Adjusted for age and sex)																	
						Univariate				Multivariate^a^					Multivariate Fully Adjusted^b^					Multivariate Fully Adjusted^c^			
*Diet Pattern*		Normal	Ovwt/Obese				95% CI					95% CI					95% CI					95% CI	
		n	n	%		OR	Lwr	Upr		OR		Lwr	Upr		OR		Lwr	Upr		OR		Lwr	Upr
		(*n* = 443)													(n=307)					(n=101)			
Healthy_C_	T1	106	45	29.8		1.00				1.00					1.00					1.00			
	T2	98	51	34.2		1.23	0.75	1.99		1.18		0.71	1.95		1.27		0.63	2.57		1.04		0.16	6.98
	T3	90	53	37.1		1.39	0.85	2.26		1.36		0.82	2.24		1.01		0.44	2.32		1.36		0.15	12.46
Junk_C_	T1	92	52	36.1		1.00				1.00					1.00					1.00			
	T2	99	52	34.4		0.93	0.58	1.50		0.94		0.58	1.54		1.01		0.47	2.18		0.47		0.08	2.75
	T3	103	45	30.4		0.77	0.47	1.26		0.80		0.49	1.32		0.44		0.18	1.06		0.62		0.08	5.05
Processed_C_	T1	105	48	31.4		1.00				1.00					1.00					1.00			
	T2	97	54	35.8		1.22	0.76	1.96		1.21		0.74	1.98		0.97		0.47	2.02		5.73		0.79	41.83
	T3	92	47	33.8		1.12	0.68	1.82		1.08		0.65	1.80		0.52		0.20	1.35		1.24		0.09	16.61
Pasta & Vegetable_C_	T1	92	53	36.6		1.00				1.00					1.00					1.00			
	T2	108	42	28		0.68	0.41	1.10		0.74		0.44		1.23	0.36	**	0.18	0.75		0.19		0.03	1.26
	T3	94	54	36.5		1.00	0.62	1.60		1.11		0.67	1.84		0.77		0.37	1.62		0.30		0.05	1.81
Cereal & Juice_C_	T1	82	61	42.7		1.00				1.00					1.00					1.00			
	T2	105	49	31.8		0.63	0.39	1.01		0.65		0.40	1.06		0.44	*	0.22	0.90		0.12	*	0.02	0.72
	T3	107	39	26.7		0.49	0.30	0.80		0.50	**	0.30	0.82		0.41	*	0.19	0.85		0.07	**	0.01	0.42

		(*n* = 435)													(*n* = 307)								
Healthy_M_	T1	89	50	36.0		1.00				1.00					1.00								
	T2	103	36	25.9		0.62	0.37	1.04		0.60		0.36	1.01		0.70		0.33	1.46					
	T3	97	60	38.2		1.10	0.69	1.77		1.02		0.63	1.68		1.37		0.63	2.98					
Processed_M_	T1	110	50	31.2		1.00				1.00					1.00								
	T2	97	58	37.4		1.32	0.83	2.10		1.30		0.79	2.12		2.64	**	1.28	5.45					
	T3	82	38	31.7		1.02	0.61	1.70		0.96		0.57	1.62		2.03		0.87	4.73					
Cereal & Fruit_M_	T1	89	43	32.6		1.00				1.00					1.00								
	T2	102	49	32.5		0.99	0.60	1.64		0.95		0.57	1.59		1.02		0.47	2.21					
	T3	98	54	35.5		1.14	0.70	1.87		1.15		0.69	1.92		1.41		0.61	3.26					
Tea & Bread_M_	T1	83	51	38.1		1.00				1.00					1.00								
	T2	101	50	33.1		0.81	0.50	1.31		0.81		0.49	1.33		1.17		0.56	2.46					
	T3	104	45	30.2		0.70	0.43	1.15		0.75		0.45	1.25		0.69		0.28	1.67					
Trad'l_M_	T1	99	59	37.3		1.00				1.00					1.00								
	T2	103	46	30.9		0.75	0.47	1.20		0.71		0.44	1.16		0.52		0.26	1.05					
	T3	87	41	32.0		0.79	0.48	1.29		0.77		0.46	1.28		0.69		0.30	1.61					
Conf'y_M_	T1	90	53	37.1		1.00				1.00					1.00								
	T2	99	47	32.2		0.81	0.50	1.31		0.80		0.48	1.33		1.13		0.55	2.33					
	T3	100	46	31.5		0.78	0.48	1.27		0.77		0.46	1.28		1.13		0.55	2.31					

		(*n* = 330)																		(*n* = 101)			
Healthy_F_	T1	76	34	30.9		1.00				1.00										1.00			
	T2	71	39	35.5		1.23	0.70	2.15		1.39		0.77	2.49							2.21		0.39	12.39
	T3	74	36	32.7		1.09	0.62	1.92		1.15		0.64	2.06							2.29		0.42	12.56
Processed_F_	T1	66	44	40.0		1.00				1.00										1.00			
	T2	73	37	33.6		0.76	0.44	1.32		0.68		0.38	1.22							0.61		0.12	3.01
	T3	82	28	25.5		0.51	0.29	0.91		0.50	*	0.28	0.90							0.74		0.14	3.98
Cereal & Fruit_F_	T1	78	32	29.1		1.00				1.00										1.00			
	T2	68	42	38.2		1.51	0.86	2.64		1.45		0.81	2.57							2.16		0.44	10.61
	T3	75	35	31.8		1.14	0.64	2.02		1.11		0.61	2.00							0.68		0.13	3.49
Tea & Bread_F_	T1	78	32	29.1		1.00				1.00										1.00			
	T2	75	35	31.8		1.14	0.64	2.02		1.11		0.62	2.01							0.60		0.12	3.06
	T3	68	42	38.2		1.51	0.86	2.64		1.49		0.84	2.67							1.00		0.21	4.70
Trad'l_F_	T1	67	43	39.1		1.00				1.00										1.00			
	T2	75	35	31.8		0.73	0.42	1.27		0.75		0.42	1.31							0.63		0.11	3.48
	T3	79	31	28.2		0.61	0.35	1.08		0.66		0.37	1.20							0.32		0.07	1.53
Conf'y_F_	T1	76	34	30.9		1.00				1.00										1.00			
	T2	71	39	35.5		1.23	0.70	2.15		1.11		0.62	1.98							2.20		0.38	12.92
	T3	74	36	32.7		1.09	0.62	1.92		0.96		0.53	1.74							1.98		0.35	11.27

Abbreviations: BMI, body mass index; CI, confidence interval; OR, odds ration. *p < 0.05, ** *p* < 0.01; **^a^** multivariate model adjusted for the diet pattern variables; **^b^** mulitvariate model adjusted for energy intake (kcal) for mother and child, child's birthweight, mother's age, BMI prepregnancy, education and smoking during pregnancy;**^c^** mulitvariate model adjusted for energy intake (kcal) for father and child, child's birthweight, father's age, BMI at baseline, education and smoking at baseline.

## 4. Discussion

This study has shown that a diet pattern scoring higher in processed foods during pregnancy, comprising of soft drinks, chips, roast potatoes, crisps, pizza, processed meat, sweets and chocolate, had a strong and significant association with offspring overweight and obesity at the age of five years. The association was apparent after adjustment for the child's diet at five, and for a range of socio-economic, lifestyle and pregnancy related variables. A similar analysis conducted with the perinatal dietary patterns of fathers did not show any association with offspring adiposity at age five. To our knowledge, this present study is the first to prospectively examine diet patterns during pregnancy with offspring adiposity in early childhood.

Six similar dietary patterns were observed for mothers and fathers during the perinatal period and these patterns were correlated between the mother-father pairs. Most studies report fewer dietary patterns as the first components tend to explain much of the variance. All six components were maintained for this analysis as similar percentage variance was explained for each pattern. The first two dietary patterns (healthy and processed) resembled other studies of diet patterns in pregnancy, often referred to as the “prudent” and “Western” diet patterns [40],[42]. Similar patterns to those reported here have also been observed in an adult Irish population; Hearty et al. identified four components described as “unhealthy foods & high alcohol”, “traditional Irish”, “healthy foods”, “sweet convenience foods & low alcohol” [69]. In a large prospective birth cohort study, healthy, processed and traditional patterns were also observed for British adults [49]. Furthermore, the correlations observed between male-female partners (*n* = 4668 couples) were analogous to the relationships we observed between parents in the Lifeways study [49].

Some of the diet patterns observed for the children in the study were similar to those reported elsewhere. The “health conscious”, “processed” and “junk” patterns were reported by ALPSAC at age 4 years and characterized by similar foods [70],[71]. Data from the UK National Diet and Nutrition Survey identified a “healthy”, “traditional” and “convenience” diet using Cluster analysis [72]. Dietary patterns have also been described for Spanish, Australian, and Finnish children but the patterns were less similar than those of the British studies [73]–[75].The five diet patterns observed for the children aged five years also showed certain correlations with their parent's diet. The “junk” diet pattern was consistently correlated with the processed pattern in both parents, while the pasta & veg pattern was correlated with the healthy pattern in both parents. The mothers' healthy pattern was correlated with the children's healthy pattern but also the children's processed pattern. The child's cereal and juice pattern was correlated with both the maternal healthy pattern and confectionery pattern. Some of these unexpected relationships may be the result of different food instruments or food groupings used for the adults and children, or they may reflect the fact that dietary patterns in the children are not always consistent with what their parents eat. Furthermore, certain food patterns could be differentially influenced by mothers or fathers, particularly at different stages of childhood; mothers may have the greatest influence during early childhood whereas fathers may become more influential later in childhood and adolescence. The relationships may also have arisen because there were fewer patterns derived for the children given the shorter food list relative to their parents. Certain food items, such as “red meat”, were grouped in the “traditional” pattern for parents but in the “processed” pattern for children. This could be explained by the fact that, in the children's FFQ, minced beef and burgers were consumed more frequently than other types of red meat.

The lack of a positive finding between children's diet pattern and overweight in this study is not uncommon as several studies have been unable to demonstrate an association [65],[76]–[78]. Other studies have identified patterns associated with obesity but these tend to be reported in older children or adolescents [79]–[81]. This may be due to the method of dietary assessment which may have excluded food items relevant to a child's diet or there may have been issues of under and over reporting of specific items. In this analysis we did not adjust for the potential effect of breastfeeding or premature weaning which could mediate the association between maternal diet and child outcomes. Equally, the use of BMI as the outcome measure of adiposity has its limitations and different reference standards and cut-offs applied in different studies can make comparisons difficult. Nevertheless, we observed a negative association for the “cereal and juice” pattern with overweight and obesity. This pattern was characterized by breakfast cereals, fruit juices and wholemeal bread. Burke et al. also demonstrated an inverse association between BMI at age eight and a “cereals” diet component (comprised of bread, cereals, spreads, and jam) and this was the only diet factor that remained statistically significant in models that included mother's BMI [75]. A recent systematic review has highlighted the positive impact of consumption of breakfast cereals in relation to obesity [82]. The diet pattern reported in the present analysis may be indicative of the importance of consumption of breakfast meals particularly in children.

The effect of the “processed” diet pattern in pregnancy was positively associated with offspring overweight and obesity. It is possible that a diet pattern which was adhered to in pregnancy could be continued post-partum and children are influenced by this type of diet in the home environment. Certainly, the correlations reported here demonstrate that certain dietary patterns are similar within families. Nevertheless, the influence of the processed pattern during pregnancy remained when adjusted for the child's diet at age five. Indeed, other birth cohorts have reported that this energy dense-type diet during pregnancy was negatively associated with foetal growth [40],[83] which may lead to increased risk of obesity in childhood. The low-nutrient, energy dense foods typical of the “processed” pattern are recognized as potential contributors to the energy imbalance that results in obesity and we have previously reported on positive associations for macronutrient intake during pregnancy that are indicative of that pattern [60]. Animal studies have allowed for the examination of possible mechanisms which mediate the impact of maternal diet on foetal development and developmental programming of obesity [6]. The studies have focused on pathways which are centrally involved in energy homeostasis including alterations in central nervous system appetite control, lasting changes in adiposity and proportions of fat and lean body mass, and pancreatic structure and function [84],[85].

In the present study, it was anticipated that there would be a clear negative relationship with the maternal “health conscious” diet pattern and offspring weight status. While the middle tertile was negatively associated with lower overweight/obesity, the highest scores for this pattern were more likely to have children with higher weights. Although the result for the highest tertile was not statistically significant the direction was unexpected as it was hypothesized that increasing scores in the pattern would be associated with reduced likelihood of overweight and obesity in offspring. This finding may have arisen due to over-reporting of fruit and vegetables for individuals which maybe introduce a bias in the upper tertile of the “health conscious” component [86]. Similarly, the upper tertile of the “processed” component may have lower overall scores due to underreporting. Some studies have reported that increasing intakes of fruit and vegetables may be associated with excessive gestational weight gain [87] and high birthweight [15],[16],[18] both of which may determine later offspring growth. However, this anomaly is likely to be as a result of the over-reporting of fruit and vegetable intake as there is a possibility that pregnant women, in particular, may be more likely to over-report “healthy foods” and underreport “unhealthy foods” in an attempt to appear to meet dietary recommendations [88].

While fathers' diet patterns were correlated with the mothers' patterns during the perinatal period, they did not appear to show any independent association with offspring adiposity. Few studies have explored dietary intakes of fathers in relation to their child's weight status. Studies have reported positive associations between the food and nutrients intakes of fathers and their children [89],[90] but none have explored patterns before the birth of their children.

The strengths of the present study include the prospective birth study design, the dietary assessment conducted during the first trimester of pregnancy and the perinatal dietary assessment of fathers. Furthermore, the study allows for the control of a range of lifestyle and socioeconomic factors which independently predict childhood obesity including maternal pre-pregnancy BMI and child birthweight.

Selection bias is an inherent issue in prospective studies and, at the outset, we identified that mothers of lower socio-economic status were less represented in the follow-up study. Nevertheless, the nutritional status of the mothers who remained in the study was not found to be significantly different in terms of the key markers of BMI and energy intake. Fathers were recruited to the study on invitation from the mother and the resulting sample size was smaller, relative to the mother. There was some degree of attrition in the follow-up of the fathers but this does not appear to have been due to a socioeconomic bias. However, the resulting sample may not have been sufficiently powered to test for multiple associations with the children, as is evidenced by some of the wide confidence intervals in the fully adjusted models, which should be interpreted with caution.

Mis-reporting of food intakes may arise with the use of FFQs however this approach has been shown to provide valid estimates of food intake during pregnancy [91] and is also comparable with weighed intakes when comparing the results of PCA [92]. The dietary pattern analysis was conducted using PCA which has the advantage of providing data driven patterns with no *a priori* assumptions. A certain level of bias may be introduced due to the number and types of food groups included in the PCA analysis. We based our approach on that used by Crozier et al given the similarity in dietary instrument used and study design [42]. However, these selections are subjective and a more robust method for grouping the foods is warranted. We attempted to reduce the bias associated with naming the components by using two researchers to independently identify the pattern descriptors. While the parental diet patterns were named in a similar way, there were differences in the loadings for certain food items, and therefore the patterns should not be considered as being identical.

Finally, while we attempted to adjust for numerous potential confounding variables, we cannot exclude the possibility of residual confounding, particularly in relation to food and lifestyle behaviours in early childhood.

## 5. Conclusion

We found a significant association between a maternal “processed” diet pattern characterized by low-nutrient, energy dense foods and offspring overweight at age five years. We also observed that children scoring highly on a cereal and juice related pattern were less likely to be overweight and obese. Further prospective studies are needed to confirm these findings.
